# Socioeconomic differences in school dropout among young adults: the role of social relations

**DOI:** 10.1186/s12889-015-2391-0

**Published:** 2015-10-15

**Authors:** Trine Nøhr Winding, Johan Hviid Andersen

**Affiliations:** Danish Ramazzini Centre, Department of Occupational Medicine, University Research Clinic, Regional Hospital West Jutland, Gl. Landevej 61, 7400 Herning, Denmark

**Keywords:** Dropout, Socioeconomic position, Social relations, Young people

## Abstract

**Background:**

School dropout in adolescence is an important social determinant of health inequality in a lifetime perspective. It is commonly accepted that parental background factors are associated with later dropout, but to what extent social relations mediate this association is not yet fully understood.

Aim: To investigate the effect of social relations on the association between parental socioeconomic position and school dropout in the Danish youth cohort Vestliv.

**Methods:**

This prospective study used data from questionnaires in 2004 and 2007 and register data in 2004 and 2010. The study population consisted of 3,054 persons born in 1989. Information on dropout was dichotomised into those who had completed a secondary education/were still attending one and those who had dropped out/had never attended a secondary education. Logistic regression analyses were used to investigate associations between parental socioeconomic position and dropout at age 21, taking into account effects of social relations at age 15 and 18.

**Results:**

A large proportion of young people were having problems with social relations at age 15 and 18. In general, social relations were strongly related to not completing a secondary education, especially among girls. For instance, 18-year-old girls finding family conflicts difficult to handle had a 2.6-fold increased risk of not completing a secondary education. Young people from low socioeconomic position families had approximately a 3-fold higher risk of not completing a secondary education compared to young people from high position families, and the estimates did not change greatly after adjustment for social relations with family or friends. Poor relations with teachers and classmates at age 18 explained a substantial part of the association between income and dropout among both girls and boys.

**Conclusions:**

The study confirmed a social gradient in completion of secondary education. Despite the fact that poor social relations at age 15 and 18 were related to dropout at age 21, social relations with family and friends only explained a minor part of the socioeconomic differences in dropout.

However, poor social relations with teachers and classmates at age 18 explain a substantial part of the socioeconomic difference in dropout from secondary education.

**Electronic supplementary material:**

The online version of this article (doi:10.1186/s12889-015-2391-0) contains supplementary material, which is available to authorized users.

## Background

Some of the strongest determinants of health are structural factors such as national wealth, income inequality, and access to education [1]. A Danish report on determinants of health inequality in a lifetime perspective points out poor educational outcome in adolescence as one of the most important of these determinants [2]. In Denmark, approximately 25 % of the 25-year-olds had not completed a secondary education in 2013 [3]. Those who do not complete a secondary education are at greater risk of developing health problems later in life [4], and across OECD countries, people with poor educational outcome are less likely to be participants in the work force [5] and are at greater risk of sickness and disability in young adulthood [6]. Furthermore, a widening of social inequality in life expectancy between those who obtained a secondary education and those who did not has been reported in Denmark in the recent years [7], indicating that dropout is indirectly related to the development of health inequality during life [2, 4].

One of the strongest risk factors of dropout is parental socioeconomic position [8–11]. Parents’ educational level, occupational prestige, and family income have been shown to have direct and indirect relationships with youths’ later educational outcome [8, 12]. Academic achievement during compulsory school has also been found to be strongly associated with dropout from secondary school [13, 14]. Previous studies have shown that parental involvement in their offspring’s schooling is an important determinant of both later academic achievement and dropout [15–17]. However, a study by Blondal et al. showed that parenting style more strongly predicts school dropout than parental involvement in school activities [18]. Apart from family relations, a good teacher-student relationship was found to be associated with lower student dropout rates [19], and close friendships were found to stimulate a sense of school belonging and academic performance among high school students [20–22], and a positive atmosphere at school increases the educational aspirations of young people [23].

Although there is some indication that adolescents’ social relations with family, friends, teachers, and classmates influence later academic achievement, the influence on school dropout has not been adequately investigated. In order to reduce social inequality, it is important to identify potential conditions that early in life mediate the relation between parental background factors and later school dropout. Identification of such mediators potentially offers important implications for prevention and intervention.

The purpose of this prospective study was to investigate the effect of social relations on the association between parental socioeconomic position and dropout from secondary education in a Danish youth cohort. Gender differences appear to play a role in the way socioeconomic measures and health are related [24]. A previous study within the Vestliv cohort showed that stress levels in girls were most strongly associated with lower parental education and that stress levels in boys were most strongly associated with parental income [24]. To evaluate the impact of the two different measures of socioeconomic position on social relations and the risk of school dropout, results were presented for each gender separately. Social relations were grouped into three different dimensions: social relations in the family, social relations with friends, and social relations at school (with classmates and teachers). To investigate the independent impact of different social environments in early and late adolescence, information about social relations was collected when the participants were 15 and 18 years old. The time between these two age points represents a very important stage of the life course, with a transition from a more family centred environment to a broader environment more open to the influence of peers and non-family members.

The following research questions were addressed: 1) Are social relations at age 15 and 18 related to dropout at age 21? 2) Is a social gradient in dropout present among 21-year-olds in Denmark? 3) Do social relations at age 15 and 18 mediate the association between parental socioeconomic position and dropout? 4) Are the relations affected by the choice of socioeconomic measure? 5) Are there gender differences in the associations between social relations, socioeconomic position and dropout from secondary education?

## Methods

### Sample

The source population of the prospective cohort study Vestliv consisted of all individuals born in 1989 and living in the county of Ringkjoebing, Denmark, in early April 2004. A total of 3,681 fulfilled these criteria, and contact information was retrieved from the Central Office of Civil Registration and from public schools in the county of Ringkjoebing. All 3,681 individuals were contacted and asked to fill out an initial questionnaire during school hours when they were 15 years of age. Those not at school on the day of collection received the questionnaire by post, resulting in a participation rate of 83 % (*n* = 3,054). Altogether 1,399 children received the questionnaire by post and 58 % completed it. A follow-up survey was conducted in 2007 when the participants were aged 18 using both e-mailed and postal questionnaires. This resulted in 2,181 participants (71 % of initial). To gather information on family socioeconomic position and dropout from secondary education, respondents were linked to their parents or guardians by using their personal identification number (CPR number), which is given to every inhabitant in Denmark at birth (or upon entry for immigrants) [25]. The study sample of the present report was defined by the 3,054 participants who answered the initial questionnaire and with available information on outcome and at least one of the exposure variables. The study was approved by the Danish Data Protection Agency.

### Measures

#### Outcome

##### Completion of secondary education

In Denmark education beyond compulsory school (secondary education) consists primarily of a high school academic track of 3 years, or vocational education, which lasts between 2 and 4 years. The outcome of the present study was completion of a secondary education after compulsory school in October 2010 when the participants were 21 years old, which allowed a follow-up of 6.5 years. Data on secondary education were based on register information derived from Statistics Denmark [26]. The Danish Education Registers collect information on all individuals attending education in Denmark and link information within and across years through the CPR number. Generally, the registers are considered of high quality [26]. The participants were categorised into those who (1) Completed/were attending: consisting of participants who had completed a secondary education or were still attending one, and (2) Dropped out/never attended: if they had dropped out of their last secondary education and never attended another or if they had never attended a secondary education.

### Exposures variables

#### Socioeconomic position

Information from registers about highest attained education in the household and household income in year 2003 was chosen as measures of socioeconomic position. Based on the source population (*N* = 3,681), yearly household income was recoded into tertiles corresponding to lowest (<61,770 EUR), middle (61,770-80,531 EUR), and highest (>80,531 EUR) [27]. Highest attained education in the household was recoded into three categories: < 10 years, 10–12 years, >12 years [26]. If the participants’ parents were divorced, information stemmed from the household at which the participants’ address was listed.

#### Social relations with parents, friends, teachers and classmates

Social relations were conceived in a general framework as having three different dimensions: 1. Social relations in the family, 2. Social relations with friends, 3. Social relations with teachers and classmates. Information about social relations was based on questionnaire information collected at age 15 and age 18. At age 15 the General Functioning Scale was used as a measure of the social climate in the family. It is made up of twelve items that assess the overall health/pathology of the family and is one of seven scales from the Family Assessment Device (FAD) [28]. Low scores indicate healthier functioning than higher scores. In this sample the mean score was 1.75, SD 0.52 and Cronbach’ alpha was 0.85. A cut-off at the 75 %-percentile (2.08) divided the scores into good/poor family functioning. As a measure of the social climate in the family at age 18, a question was asked about whether it is difficult to handle conflicts in the family (yes, sometimes or often vs. no).

Social relations with friends were measured by questions at age 15 and 18 about (1) having at least one friend to be confidential with (yes vs. no); (2) talking to friends about personal worries (very often, often or sometimes vs. not so often or rarely); (3) being satisfied with the help and support they get from friends (very often, often or sometimes vs. not so often or rarely); and (4) whether handling conflicts with friends or partner is difficult (no vs. yes, sometimes or often) [29, 30].

Social relations with teachers and classmates at age 15 and 18 were measured by questions on whether (1) teachers help with school work when it is needed (strongly agree or agree vs. disagree or strongly disagree); (2) classmates are doing well together (always, mostly or sometimes vs. rarely or never); (3) they feel left out by the other pupils in the class (always, mostly or sometimes vs. rarely or never); (4) feel attached to the classmates (strongly agree, partially agree or neither agree nor disagree vs. partially disagree or strongly disagree); or if (5) teachers help with personal problems if it is needed (strongly agree, partially agree or neither agree nor disagree vs. partially disagree or strongly disagree) [30, 31].

### Statistical analyses

A correlation analysis between measures of social relations from each time point was performed initially and no correlation exceeded 0.30 (2004) or 0.35 (2007).

Some indication of effect modification between social relations and gender was seen. Of the 13 measures of social relations 5 showed significant interactions with gender. At age 15 it was: talking to friends about personal worries, *p* = 0.001; being satisfied with the help and support they get from friends, *p* = 0.04; feeling left out by other pupils in the class, *p* = 0.02, and at age 18 it was: finding it difficult to handle conflicts with friends or partner, *p* = 0.02; feeling attached to classmates, *p* = 0.04. Gender-specific descriptive data are presented for dropout, socioeconomic position and social relations at age 15 and 18. Chi-square-tests were performed to test for gender differences.

Multiple logistic regression analyses were performed to examine gender-specific associations between socioeconomic position, different aspects of social relations and school dropout [32]. The risk estimates were odds ratios and because the prevalence’s of the social problems were high the odds ratios would tend to be skewed to a higher level compared to relative risks [33].

We first modelled how aspects of social relations were associated with not completing a secondary education (Table 2). Then we modelled the simultaneous effects of socioeconomic position and social relations on completion of secondary education after adjusting for age on completion of 9^th^ grade (Table 3). Adjustments for social relations were done for age 15 and age 18 separately because observations at the two time points were correlated.

All analyses were carried out in STATA statistical package (V.12.0; State, College Station, TX,USA).

## Results

Interactions between measures of socioeconomic position and measures of social relations were tested, but none of the tests showed a significant contribution of the interaction terms.

Table 1 shows the prevalence of aspects of completion of secondary education, social relations, and the distribution of family socioeconomic position, all together and for girls and boys, separately. Nine percent of the young people had never attended or had dropped-out of the last attended secondary education at the age of 21. A relatively large proportion of young people had problems with relations with family, friends, teachers, or classmates at the age of 15 and 18. At age 15, more boys than girls reported not having a friend to be confidential with (13 % vs. 8 %) and 46 % of the boys reported difficulties in talking to friends about personal worries, compared to 14 % of the girls. More girls than boys felt left out by other pupils in the class (16 % vs. 11 %). At age 18, more girls than boys experienced difficulties in handling family conflicts (43 % vs. 37 %) and conflicts with friends or partner (43 % vs. 37 %). At age 18, 32 % did not feel that teachers helped with personal problems if they needed it.

**Table 1:** Completed a secondary education (age 21), socioeconomic position (age 15) and social relations (age 15 and 18) all together and by gender (*n* = 3054)

	All (*n* = 3054)		Girls (*n* = 1536)		Boys (*n* = 1518)		
	n	%	n	%	n	%	*p*-value^*^
Completion of secondary education	3054		1536		1518		
Completed/attending	2779	91	1398	91	1381	91	
Dropped out/never attended	275	9	138	9	137	9	0.969
Socioeconomic position							
Household income	3053		1535		1518		
high	1092	36	541	35	551	36	
medium	1060	35	521	34	539	36	
low	901	30	473	31	428	28	0.279
missing	1		1		0		
Highest education in the household	3001		1508		1493		
high	1094	36	518	34	576	39	
medium	1548	52	801	53	747	50	
low	359	12	189	13	170	11	0.053
missing	53		28		25		
Social relations (age 15)							
Family							
Family functioning	2912		1465		1447		
very good/good	2153	74	1079	74	1074	74	
less good/not good	759	26	386	26	373	26	0.726
missing	142		71		71		
Friends							
At least one friend to be confidential with	3027		1524		1503		
yes	2700	89	1396	92	1304	87	
no	327	11	128	8	199	13	<0.001
missing	27		12		15		
Talk to friends about personal worries	3018		1521		1497		
yes	2114	70	1307	86	807	54	
no	904	30	214	14	690	46	<0.001
missing							
Satisfied with help and support from friends	3021		1522		1499		
yes	2782	92	1422	93	1360	91	
no	239	8	100	7	139	9	0.006
missing	33		14		19		
Teachers and classmates							
Teachers help me with school work when I need it	3019		1519		1500		
yes	2544	84	1263	83	1281	85	
no	475	16	256	17	219	15	0.089
missing	35		17		18		
Classmates are doing well together	3015		1520		1495		
yes	2885	96	1443	95	1442	96	
no	130	4	77	5	53	4	0.040
missing	39		16		23		
Feel left out by the other pupils in the class	3004		1515		1489		
no	2597	86	1269	86	1328	89	
yes	407	14	246	16	161	11	<0.001
missing	50		21		29		
Social relations (age 18)							
Family							
Difficult to handle conflicts	2130		1161		969		
no	1277	60	662	57	615	63	
yes	853	40	499	43	354	37	0.002
missing	924		375		549		
Friends							
At least one friend to be confidential with	2165		1173		992		
yes	2011	93	1104	94	907	91	
no	154	7	69	6	85	9	0.015
missing	889		363		526		
Difficult to handle conflicts with friends or partner	2130		1161		969		
no	1265	59	657	57	608	63	
yes	865	41	504	43	361	37	
missing	924		375		549		0.004
Teachers and classmates							
Feel attached to my classmates	1922		1065		857		
yes	1747	91	965	91	782	91	
no	175	9	100	9	75	9	0.629
missing	1132		471		661		
Teachers help me with schoolwork when I need it	1919		1064		855		
yes	1792	93	999	94	793	93	
no	127	7	65	6	62	7	0.317
missing	1135		472		663		
Teachers help me with personal problems if I need it	1911		1059		852		
yes	1292	68	709	67	583	68	
no	619	32	350	33	269	32	
missing	1143		477		666		0.493

^*^chi-square-tests were used to test for differences in completion of secondary education, socioeconomic position and social relations

### Socioeconomic differences in social relations

In general, poor socioeconomic position was related to poor social relations with family, friends, teachers, and classmates. Individuals from families with low income or low educational level more often reported poor family functioning and experienced less help and support from friends than their peers at age 15 (ORs between 1.61 and 2.05). Girls from low socioeconomic position families often reported not having a friend to be confidential with, especially at age 18 (low household income: OR 3.12 (95 % CI 1.70–5.71) and low educational level in the family: OR 3.23 (95 % CI 1.417.41)) [see Additional file 1].

### Social relations and not completing a secondary education

Social relations with family, friends, teachers, and classmates in general were strongly associated with not completing a secondary education, especially among girls (Table 2). For instance, not being satisfied with help and support from friends at age 15 was strongly associated with not completing a secondary education, especially among the girls (OR 3.02 (95 % CI 1.80–5.07), boys OR 1.73 (95 % CI 1.03–2.91)). Classmates not doing well together at age 15 was strongly related to not completing a secondary education in both girls and boys (girls: OR 3.82 (95 % CI 2.20–6.63), boys: OR 2.14 (95 % CI 1.02–4.48)). 18-year-old girls experiencing family conflicts difficult to handle had a 2.6-fold increased risk of not completing a secondary education (girls: OR 2.59 (95 % CI 1.57–4.27), boys: OR 1.34 (95 % CI 0.73–2.47)) compared to those not experiencing family conflicts difficult to handle.

**Table 2:** Odds ratios for not completing a secondary education by social relations at age 15 and 18, *n* = 3054

		Not completed a secondary education				
		All		Girls		Boys
	OR	95 %-CI	OR	95 %-CI	OR	95 %-CI
Social relations (age 15)						
Family						
Poor family functioning	1.8	1.43 ; 2.45	1.98	1.36 ; 2.88	1.77	1.21 ; 2.60
Friends						
No friend to be confidential with	1.48	1.04 ; 2.12	1.89	1.12 ; 3.18	1.23	0.75 ; 2.01
Do not talk to friends about personal worries	1.54	1.19 ; 1.99	2.41	1.60 ; 3.65	1.27	0.89 ; 1.81
Not satisfied with help and support from friends	2.24	1.55 ; 3.23	3.02	1.80 ; 5.07	1.73	1.03 ; 2.91
Teachers and classmates						
Teachers do not help me with school work when I need it	1.45	1.06 ; 1.98	1.74	1.15 ; 2.64	1.15	0.71 ; 1.87
Classmates are not doing well together	3.03	1.95 ; 4.69	3.82	2.20 ; 6.63	2.14	1.02 ; 4.48
Feel left out by the other pupils in the class	1.91	1.40 ; 2.61	2.48	1.67; 3.70	1.32	0.78 ; 2.23
Social relations (age 18)						
Family						
Difficult to handle conflicts	2.02	1.39 ; 2.96	2.59	1.57 ; 4.27	1.34	0.73 ; 2.47
Friends						
No friend to be confidential with	0.91	0.44 ; 1.90	1.43	0.60 ; 3.42	0.46	0.11 ; 1.94
Difficult to handle conflicts with friends or partner	1.83	1.26 ; 2.67	1.96	1.21 ; 3.21	1.57	0.86 ; 2.88
Teachers and classmates						
Do not feel attached to my classmates	1.92	0.93 ; 3.98	1.92	0.78 ; 4.73	1.88	0.54 ; 6.55
Teachers do not help me with schoolwork when I need it	1.37	0.54 ; 3.50	1.33	0.40 ; 4.45	1.52	0.34 ; 6.74
Teachers do not help me with personal; problems if I need it	0.61	0.33 ; 1.44	0.53	0.24 ; 1.17	0.77	0.27 ; 2.16

### Socioeconomic position, social relations, and not completing a secondary education

Table 3 shows that young people from the lowest socioeconomic position families had approximately a 3-fold higher risk of not completing a secondary education compared to young people from the highest socioeconomic position families (Model 1), and a significant trend was seen across socioeconomic groups. Socioeconomic differences in completion of secondary education did not change substantially after adjustment for family relations (Models 2 and 6) or relations with friends (Models 3 and 7).

**Table 3:** Odds ratios for not completing a secondary education by parents socioeconomic position (Model 1), controlled for social relations with family (Model 2 and 6), friends (Model 3 and 7), and teachers and classmates (Model 4 and 8) and for all social relations (Model 5 and 9) in 2004 or 2007 (*n* = 3054)

Not completed a secondary education										
	All				Girls			Boys		
Model 1	OR	95 %-CI		*P*-value	OR	95 %-CI	*P*-value	OR	95 %-CI	*P*-value*
Income										
	high	ref			ref			ref		
	medium	1.47	1.03 ;2.09		1.54	0.93 ; 2.54		1.40	0.85 ; 2.30	
	low	3.09	2.23 ; 4.27	<0.001	2.86	1.80 ; 4.54	<0.001	3.29	2.08 ; 5.19	<0.001
Highest education										
	high	ref			ref			ref		
	medium	1.28	0.94 ; 1.75		1.02	0.66 ; 1.59		1.61	1.04 ; 2.48	
	low	3.11	2.15 ; 4.49	<0.001	2.70	1.62 ; 4.51	<0.001	3.51	2.07 ; 5.97	<0.001
Adjusted for social factors at age 15										
Model 2										
Income										
	high	ref			ref			ref		
	medium	1.39	0.97 ; 1.99		1.46	0.87 ; 2.45		1.32	0.79 ; 2.21	
	low	2.82	2.01 ; 3.94	<0.001	2.67	1.66; 4.31	<0.001	2.96	1.84 ; 4.76	<0.001
Highest education										
	high	ref			ref			ref		
	medium	1.25	0.91 ; 1.72		0.96	0.61 ; 1.51		1.62	1.03 ; 2.55	
	low	3.05	2.08 ; 4.47	<0.001	2.56	1.51 ; 4.35	<0.001	3.57	2.05 ; 6.21	<0.001
Model 3										
Income										
	high	ref			ref			ref		
	medium	1.40	0.98 ; 2.01		1.43	0.86 ; 2.37		1.35	0.81 ; 2.26	
	low	3.07	2.21 ; 4.28	<0.001	2.74	1.71 ; 4.37	<0.001	3.40	2.12 ; 5.44	<0.001
Highest education										
	high	ref			ref			ref		
	medium	1.31	0.96 ; 1.80		1.00	0.64 ; 1.56		1.71	1.09 ; 2.68	
	low	3.06	2.10 ; 4.45	<0.001	2.44	1.44 ; 4.13	<0.001	3.72	2.16 ; 6.42	<0.001
Model 4										
Income										
	high	ref			ref			ref		
	medium	1.45	1.02 ; 2.08		1.47	0.89 ; 2.44		1.44	0.87 ; 2.39	
	low	2.80	2.01 ; 3.91	<0.001	2.44	1.52 ; 3.93	<0.001	3.22	2.02 ; 5.15	<0.001
Highest education										
	high	ref			ref			ref		
	medium	1.31	0.96 ; 1.80		1.08	0.69 ; 1.71		1.60	1.03 ; 2.49	
	low	3.15	2.16 ; 4.59	<0.001	2.64	1.55 ; 4.51	<0.001	3.72	2.17 ; 6.36	<0.001
Model 5										
Income										
	high	ref			ref			ref		
	medium	1.37	0.95 ; 1.99		1.30	0.77 ; 2.21		1.41	0.83 ; 2.40	
	low	2.67	1.88 ; 3.78	<0.001	2.31	1.41 ; 3.78	0.002	3.10	1.88 ; 5.10	<0.001
Highest education										
	high	ref			ref			ref		
	medium	1.29	0.92 ; 1.79		0.94	0.59 ; 1.51		1.72	1.07 ; 2.77	
	low	3.07	2.07 ; 4.56	<0.001	2.24	1.28 ; 3.91	0.003	4.03	2.27 ; 7.14	<0.001
Adjusted for social factors at age 18										
Model 6										
Income										
	high	ref			ref				ref	
	medium	1.76	1.04 ; 2.99		1.68	1.28 ; 3.11		1.91	0.83 ; 4.39	
	low	3.42	2.06 ; 5.69	<0.001	3.56	2.01 ; 6.29	<0.001	3.31	1.46 ; 7.50	0.015
Highest education										
	high	ref			ref			ref		
	medium	1.38	0.87 ; 2.20		0.97	0.54 ; 1.73		2.80	1.19 ; 6.58	
	low	3.76	2.14 ; 6.61	<0.001	2.43	1.20 ; 4.95	0.018	8.18	3.05 ; 21.97	<0.001
Model 7										
Income										
	high	ref			ref			ref		
	medium	1.77	1.04 ; 3.01		1.70	0.85 ; 3.39		1.94	0.84 ; 4.46	
	low	3.43	2.06 ; 5.71	<0.001	3.40	1.76 ; 6.56	<0.001	3.32	1.46 ; 7.53	0.015
Highest education										
	high	ref			ref			ref		
	medium	1.40	0.88 ; 2.24		0.99	0.55 ; 1.77		2.79	1.19 ; 6.58	
	low	3.92	2.23; 6.88	<0.001	2.50	1.23 ; 5.10	0.016	8.30	3.08 ; 22.32	<0.001
Model 8										
Income										
	high	ref			ref			ref		
	medium	1.13	0.59 ; 2.18		1.23	0.54 ; 2.80		0.95	0.31 ; 2.86	
	low	1.51	0.76 ; 2.97	0.484	1.52	0.64 ; 3.61	0.640	1.42	0.46 ; 4.36	0.755
Highest education										
	high	ref			ref			ref		
	medium	0.90	0.47; 1.71		0.65	0.29 ; 1.45		1.55	0.51 ; 4.70	
	low	3.37	1.60 ; 7.10	<0.001	2.50	1.01 ; 6.14	0.014	5.28	1.34 ; 20.83	0.051
Model 9										
Income										
	high	ref			ref			ref		
	medium	1.07	0.54 ; 2.10		1.12	0.47 ; 2.67		0.90	0.29 ; 2.73	
	low	1.44	0.72 ; 2.90	0.548	1.50	0.61 ; 3.70	0.661	1.34	0.43 ; 4.15	0.785
Highest education										
	high	ref			ref			ref		
	medium	0.85	0.44 ; 1.64		0.61	0.26 ; 1.39		1.51	0.50 ; 4.61	
	low	2.98	1.37 ; 6.47	0.003	2.18	0.84 ; 5.65	0.036	6.03	1.50 ; 24.33	0.033

All models are adjusted for age when completing 9^th^ grade

* Test for trend

Adjusting for social relations with classmates and teachers at age 18 reduced the association between family income and the chance of completing a secondary education (OR changed from 3.09 (95 % CI 2.23–4.27) to 1.51 (95 % CI 0.76–2.97)) (Model 8).

In general, the large socioeconomic differences in young people’s chance of completing a secondary education remained after simultaneous adjustments for all social relations, both at age 15 (Model 5) and age 18 (Model 9). However, adjusting for all social relations at age 18 reduced the strength of the association between family income and the chance of completing a secondary education considerably. The odds ratio changed from 3.09 (95 % CI 2.23–4.27) in the crude analysis to 1.44 (95 % CI 0.72–2.90) in the fully adjusted analysis, but this was not the case when adjusting for social factors from age 15 (OR changed to 2.67 (95 % CI 1.88–3.78). The associations between family educational level and the chance of completing a secondary education remained strong after adjustment for all social relations both at age 15, Model 5 (OR 3.07 (95 % CI 2.07–4.56)) and age 18, Model 9 (OR 2.98 (95 % CI 1.37–6.47)).

## Discussion

The present study showed that poor social relations with parents, friends, teachers, and classmates are common among 15- and 18-year-old Danish adolescents. Among both girls and boys, the risk of not having completed a secondary education at age 21 increased if an individual had experienced poor social relations, but at the same time poor social relations with family and friends only explained a minor part of the socioeconomic differences in dropout from secondary education. Poor social relations with teachers and classmates at age 18 explained a large part of the association between income and dropout among both girls and boys.

Most previous research on the influence of social relations on educational outcomes has focused on parent’s investment and involvement in their children’s school, and parental interest appears to facilitate the offspring’s motivation for schoolwork and improve both academic achievement and adult educational outcome [8, 16, 34]. Henry et al. reported parental investment in school as a mediator of the relationship between socioeconomic status and students’ expectation to graduate from high school [8], but they did not investigate whether the students succeeded in graduating or not. On the other hand, a study by Blondal et al. found that parenting style at age 14 was a stronger predictor than parental involvement in terms of having completed upper secondary school by age 22 [18]. One of the strengths of the study by Blondal et al. is that it like the present study, included social relations from different social environments.

Some gender differences were found in the current study. The associations between parental socioeconomic position and dropout were strong in both genders, and especially among the boys, which is consistent with previous findings [9, 35]. At the same time, poor social relations were more strongly associated with not completing a secondary education among girls than among boys. This finding stresses the importance of parents, teachers, and other adults being in contact with adolescent girls to help stimulate positive social relations.

Other studies have confirmed strong associations between negative relations with parents [12, 36] friends, teachers, and classmates [19–22] and lack of educational outcomes in their children but only a few studies have evaluated the influence of poor social relations on the association between socioeconomic position and dropout. A previous study documented that in addition to lower socioeconomic position being related to school dropout, students from lower socioeconomic families were generally more disengaged in school than students from higher socioeconomic families [37]. In addition Melby et al. found that family income of 7^th^ grade students has both a direct and an indirect effect on educational attainment through supportive parenting [12]. Whether the positive effect of social relations on educational outcome is due to increased school motivation and engagement among the students needs further investigation.

Test for trends overall showed a clear dose–response pattern between level of household income or highest education in the household and completion of secondary education of the young people. The only tests not being statistically significant were between income level and school completion after adjustment for social factors at age 18 (Models 8 and 9).

Previous research suggests that different measures of socioeconomic position, such as parental income and education, affect health and future social status through different pathways [38]. Bourdieu differentiates between two independent yet interrelated mechanisms: economic capital (income) and cultural capital (educational level). He argues that having low levels of economic capital could make a person more prone to living in situations that are more stressful, e.g. lack of material resources, whereas low levels of cultural capital would influence the way a person copes with stressful situations [39]. By including highest education in the household and household income as two separate exogenous variables, we were able to evaluate the contribution of each socioeconomic component. We found both measures related to dropout in young adulthood, but the results indicate that they are related in slightly different ways and that the mechanisms to some extent vary by gender. In general, parental educational level (cultural capital) appeared to have a larger influence on boys’ chances of completing a secondary education than household income (economic capital) when social relations were taken into account, whereas among girls, no clear pattern was observed. This finding is in line with the results of a study in a Norwegian male population [40].

In the present study, poor social relations with teachers and classmates at age 18 seemed to explain part of the socioeconomic difference in dropout. Actually, it seemed that social relations with teachers and classmates were mediators of the association between household income and completion of a secondary education but not between parental educational level and completion of secondary education. The reason for the difference between the estimates of the two socioeconomic measures is not obvious. However, the results indicate that the importance of social relations at school increases from age 15 to 18 concurrently with the natural transition during adolescence, especially among young people from stressful environments due to low economic capital. It seems that late adolescence is an important stage of the life course, with a transition from a strong parental influence to greater influence of classmates, teachers, and other non-family members.

This study features a relatively high initial participation rate of 83 % of whom 71 % responded again at follow-up in 2007. Additional strengths of the study are the prospective design with complete follow-up due to use of register-based data. At the same time, the use of both questionnaire and register-based data minimises the risk of common method variance [41].

It is important to emphasise that the questions asked about social relations with classmates and teachers at the two different age points are not all identical. As such, the difference between social relations’ mediating role at ages 15 and 18 might be attributable to the different constructs that were measured rather than the age periods per se.

Some of the missing answers to the questions about social relations at age 18 could be due to school dropout prior to this age. Altogether 147 participants reported being out of school when they completed the first follow-up questionnaire at age 18. This selection problem could result in bias due to missing information from some of the adolescents with highest risk of negative educational outcome. However, it is not clear how this missing information may have influenced the results.

The high frequency of young people attending or having completed a secondary education (91 %) by age 21 indicates that some selection into the Vestliv cohort has occurred. A previous study on the same data material demonstrated that the participants had slightly better school abilities and more often came from homes with two adults, higher income, or higher educational level. These differences increased at subsequent follow-ups. Although certain characteristics were related to those who participate initially and at follow-ups, this did not have any large influence on the relative risk estimates measured in the study. This is reassuring for the validity of the relative estimates in the current study [42].

Social relations with family, friends, teachers, and classmates in general only explained a small part of the association between socioeconomic position and dropout. It is likely that other aspects such as major life events like death or illness in the family, divorce, or living with one parent could potentially influence the chance of completion as well. Including such variables in future studies is recommended.

The objective of this study was not to study social inequality of health per se but to address some potential determinants that eventually could lead to poor health outcome. Addressing inequality in young people’s educational outcome has multiple potential benefits that extend beyond reductions in health inequalities. If this inequality could be reduced, it would enable young people to maximise their capabilities and eventually be able to participate equally with others in society. Given the relatively low social inequality in Denmark, the results can be difficult to generalise to other more unequal countries. However, the fact that the difference in life expectancy between those who complete secondary education and those who do not is increasing in Denmark [7], indicates that positive social relations that are preventing school dropout is indirectly related to the prevention of health inequality later in life [2, 4].

## Conclusion

This study confirmed a social gradient in completion of secondary education among Danish students. Despite the fact that poor social relations at age 15 and 18 were related to dropout at age 21, social relations to family and friends only explained a minor part of the socioeconomic differences in dropout from secondary education. However, poor social relations with teachers and classmates at age 18 explain a substantial part of the socioeconomic difference in dropout from secondary education. The findings suggest that stimulating positive social relations with classmates and teachers may benefit all students and could potentially reduce the risk of adolescents from economically disadvantaged families not getting a secondary education, which may be a part of a number of life events that eventually could lead to social and health inequality.

## Additional file

Additional file 1:
**Description of data: Odds ratios for poor relations with parents, friends, teachers, and classmates at ages 15 and 18 by household income or highest education in the household (high vs. low) at age 15, all together and by gender,** ***n*** **= 3,054.** (PDF 19 kb)
