# Supplementary material for: Socioeconomic differences in school dropout among young adults: the role of social relations
Source: BMC Public Health. 2015 Oct 15;15:1054. doi: 10.1186/s12889-015-2391-0 (PMC4606900; doi:10.1186/s12889-015-2391-0)
Supplement: Additional file 1: — Description of data: Odds ratios for poor relations with parents, friends, teachers, and classmates at ages 15 and 18 by household income or highest education in the household (high vs. low) at age 15, all together and by gender, n = 3,054. (PDF 19 kb) [file 12889_2015_2391_MOESM1_ESM.pdf]

Additional file 1.

Odds ratios for poor relations with parents, friends, teachers and classmates at age 15 and 18 by household income (high vs. low) at age 15, alltogether and by gender, n=3054

	Household income					
	All		Girls		Boys	
**Social relations (age 15)**	OR	95%-CI	OR	95%-CI	OR	95%-CI
**Family**						
Poor family functioning	1.67	1.36 ; 2.04	1.45	1.09 ; 1.93	1.93	1.44 ; 2.59
**Friends**						
No friend to be confidential with	1.21	0.91 ; 1.61	1.62	1.03 ; 2.55	1.01	0.70 ; 1.48
Do not talk to friends about personal worries	1.20	0.99 ; 1.46	1.42	0.98 ; 2.06	1.25	0.96 ; 1.61
Not satisfied with help and support from friends	1.61	1.15 ; 2.25	1.34	0.79 ; 2.28	1.87	1.21 ; 2.90
**Teachers and classmates**						
Teachers do not help me with school work when I need it	1.29	1.01 ; 1.66	1.49	1.07 ; 2.09	1.07	0.74 ; 1.55
Classmates are not doing well together	1.87	1.21 ; 2.90	1.44	0.82 ; 2.55	2.68	1.32 ; 5.42
Feel left out by the other pupils in the class	1.61	1.24 ; 2.10	1.53	1.09 ; 2.16	1.70	1.13 ; 2.56
**Social relations (age 18)**						
**Family**						
Difficult to handle conflicts	1.44	1.15 ; 1.80	1.53	1.13 ; 2.05	1.34	0.95 ; 1.87
**Friends**						
No friend to be confidential with	1.90	1.26 ; 2.87	3.12	1.70 ; 5.71	1.15	0.64 ; 2.10
Difficult to handle conflicts with friends or partner	1.54	1.23 ; 1.92	1.70	1.26 ; 2.29	1.34	0.96 ; 1.88
**Teachers and classmates**						
Do not feel attached to my classmates	1.81	1.20 ; 2.74	2.58	1.47 ; 4.52	1.16	0.62 ; 2.19
Teachers do not help me with schoolwork when I need it	1.33	0.84 ; 2.10	1.12	0.58 ; 2.20	1.57	0.83 ; 2.96
Teachers do not help me with personal problems if I need it	0.87	0.67 ; 1.11	0.90	0.65 ; 1.25	0.82	0.55 ; 1.21

Odds ratios for poor relations with parents, friends, teachers and classmates at age 15 and 18 by highest education in the household (high vs. low) at age 15, alltogether and by gender, n=3054

	Highest education in the household					
	All		Girls		Boys	
**Social relations (age 15)**	OR	95%-CI	OR	95%-CI	OR	95%-CI
**Family**						
Poor family functioning	1.72	1.31 ; 2.24	1.98	1.36 ; 2.87	1.48	1.00 ; 2.18
**Friends**						
No friend to be confidential with	1.18	0.80 ; 1.73	1.38	0.76 ; 2.49	1.10	0.66 ; 1.83
Do not talk to friends about personal worries	1.44	1.12 ; 1.86	1.92	1.22 ; 3.03	1.55	1.10 ; 2.19
Not satisfied with help and support from friends	2.05	1.38 ; 3.04	2.22	1.23 ; 4.02	1.98	1.17 ; 3.37
**Teachers and classmates**						
Teachers do not help me with school work when I need it	1.31	0.96 ; 1.79	1.11	0.72 ; 1.70	1.55	0.98 ; 2.44
Classmates are not doing well together	1.54	0.91 ; 2.61	1.66	0.84 ; 3.29	1.33	0.58 ; 3.05
Feel left out by the other pupils in the class	1.58	1.14 ; 2.18	1.58	1.04 ; 2.39	1.46	0.86 ; 2.48
**Social relations (age 18)**						
**Family**						
Difficult to handle conflicts	1.47	1.08 ; 2.01	1.56	1.03 ; 2.35	1.29	0.79 ; 2.11
**Friends**						
No friend to be confidential with	1.39	0.77 ; 2.50	3.23	1.41 ; 7.41	0.67	0.25 ; 1.75
Difficult to handle conflicts with friends or partner	1.25	0.91 ; 1.71	1.37	0.91 ; 2.07	1.03	0.63 ; 1.70
**Teachers and classmates**						
Do not feel attached to my classmates	1.04	0.58 ; 1.86	1.35	0.67 ; 2.69	0.54	0.16 ; 1.83
Teachers do not help me with schoolwork when I need it	2.29	1.23 ; 4.24	3.96	1.80 ; 8.72	0.89	0.26 ; 3.07
Teachers do not help me with personal problems if I need it	0.67	0.46 ; 0.98	0.59	0.36 ; 0.95	0.81	0.44 ; 1.47
